# Appliance of domiciliary nutrition instruction method for the elderly people requiring long-term care in Japan

**DOI:** 10.1186/1753-6561-6-S3-P50

**Published:** 2012-06-01

**Authors:** Koko Rusu-Udagawa, Toshikazu Suzuki, Yukie Yanagisawa

**Affiliations:** 1 Graduate School of Human Ecology, Wayo Women’s University, Ichikawa, Chiba 272-8533, Japan; 2 Department of Health and Nutrition, Wayo Women’s University, Ichikawa, Chiba 272-8533, Japan

## Background

In Japan, elderly people aged 65 and over account for more than one fifth of the nation’s population. To add to this, the number of aged households is increasing year by year. Because elderly people are preferential to have tender and non-fatty foods in general, they are prone to biased nutrition status. According to the result of a National Health and Nutrition Survey, the low weight male who aged 60-69 and over percentage has increased from 7.2% (1987) to 12.3% in 2009. Also, female is 18.6% to 22.3%. Therefore both male and female has increased about 5%. lt is predicted that nutritional management for elderly person requiring long-term care but living in their home may become difficult. Establishing the novel nutritional guidance for such elderly person with their favor is one of the most important challenges for clinical dietitian in Japan. In this study, we carried out two questionnaire surveys, in order to understand nutritional status of elderly people requiring long-term care but living in their home and find out dietary preference of elderly people.

## Materials and methods

In order to grasp the present condition of care of elderly people requiring long-term care, we performed the questionnaire by mail to 197 nursing-care-services entrepreneurs in the part of Tokyo. ln order to be and to grasp the present condition of house elderly people’s meal, both the part of Tokyo and one of city in the Chiba used semi-quantitative food frequency questionnaire (FFQ) method which set the elderly people aged 65 and over as the 147-person object. Also, we performed the questionnaire about the cooking method also went simultaneously.

## Results

According to the nursing-care-services entrepreneur’s questionnaire, there was a problem as decrease of dietary intake which is 78.0%, decrease of weight of 39.6%, malnutrition of 54.9%. According to FFQ, it was characteristic that people intake of fish and shellfishes more than meat, also soft food, such as a sweet roll. Moreover, in investigation of the cooking method, there was much ingestion of the sliced raw fish, and it was simple cooking method, people would rather cook as bake.

## Conclusions

When the decrease of dietary intake is seen in connection with aging, it is necessary to recommend food with high nutrient density. Moreover, it is also important to propose the meal content which took dysphagia function into consideration enough. It is important that dietitian recommends simple recipes using microwave oven to elderly people and their family, rather than telling recipes for elaborate meals.

